# Categorizing and characterizing intestinal dysbiosis: evaluating stool microbial test clinical utility

**DOI:** 10.3389/frmbi.2025.1512257

**Published:** 2025-05-19

**Authors:** Lia Oliver, Marta Malagón, Sara Ramió-Pujol, Mireia Sánchez-Vizcaino, Roser Sánchez-Algans, Eva Lacosta, Marc Saéz-Zafra, Mariona Serra-Pagès, Xavier Aldeguer, Jesús Garcia-Gil, Sara Oduber

**Affiliations:** ^1^ GoodGut Sociedad Limitada Unipersonal (S.L.U), Girona, Spain; ^2^ Research Group on Statistics, Econometrics and Health (GRECS), University of Girona, Girona, Spain; ^3^ Centre mèdic de neurologia i psicologia (NEUPSI)-Clínica Bofill, Girona, Spain; ^4^ Biology Department, University of Girona, Girona, Spain

**Keywords:** gut microbiota, dysbiosis, clinical utility, stool test, healthy microbiome

## Abstract

**Background:**

Interest in the intestinal microbiota has surged in recent years, leading to the development of various microbiota tests utilizing stool analysis. This study aimed to assess the clinical utility of the TestUrGut.

**Results:**

The abundances of different microbial markers analyzed correlated with various factors and symptoms. While no age differences were observed, an increase in *A. muciniphila* abundance was noted in women compared to men. Body mass index significantly influenced the abundance of *A. muciniphila* and *M. smithii*. Additionally, variations in the abundances of *A. muciniphila* and *M. smithii*, as well as a greater presence of Firmicutes or Bacteroidetes based on stool patterns, were linked to diarrhea or constipation. The dysbiosis index was validated, distinguishing between temporary and pathological dysbiosis.

**Conclusions:**

This study revealed significant relationships between the intestinal microbiota and digestive tract symptoms. Microbial markers have emerged as robust indicators of the overall state of the intestinal microbiota, demonstrating that variations are closely associated with patients’ clinical symptoms.

## 1 Background

The human gut microbiota, a complex ecosystem of trillions of microorganisms, plays a crucial role in maintaining human health. Emerging evidence has demonstrated the significance of the relationship between the gut microbiota composition and various aspects of human health, including immune function, metabolism, mental health, and gastrointestinal disorders. Dysbiosis, characterized by an imbalance or perturbation in the gut microbial community, has been associated with various abnormal conditions, such as inflammatory bowel disease (IBD), irritable bowel syndrome (IBS), obesity, diabetes, and cardiovascular diseases, and is implicated in the development and progression of these conditions (Petersen and Round, 2014; Wei et al., 2021).

Regulating the gut microbiome has emerged as a promising therapeutic approach for managing chronic diseases that significantly burden healthcare systems (Chen et al., 2019). Consequently, there has been a surge of interest in developing microbial stool tests that can accurately assess gut dysbiosis and provide insights into its implications for human health.

In recent years, extensive research has been dedicated to characterizing the diversity and functional capacity of the gut microbiota, leading to the development of numerous microbial stool tests for dysbiosis assessment (Eck et al., 2017; Huang and W, 2021). However, the clinical applicability of these tests hinges on several factors, including the clinical and analytical validity of the assay, the interpretation of results by clinicians, and the successful translation of test outcomes into effective treatment strategies (Chen et al., 2019). The challenges associated with applying microbial stool tests in regular clinical practice have limited their widespread use. Factors such as the substantial volume of data generated from microbiome tests, the considerable interindividual variation in gut microbial composition, and the lack of disease condition-specific microbial profiles have hindered the seamless integration of these tests into routine clinical workflows (Bäckhed et al., 2012; Oh and Rezaie, 2022).

Efforts are being made to overcome these challenges by improving the clinical validity of microbial stool tests, standardizing interpretation guidelines, and establishing disease-specific microbial signatures. These advancements aim to enhance the practical applicability of gut microbiome information, ultimately facilitating its effective utilization in clinical decision-making and personalized treatment approaches (Tiffany and Bäumler, 2019; Wei et al., 2021).

Nevertheless, the definition of a “healthy” gut microbiome remains a challenge, rendering the accurate determination of dysbiosis even more complex. The gut microbiota composition is highly individualized and influenced by various factors, including genetics, diet, lifestyle, and environmental exposures. Considerable interindividual variation in microbial diversity and abundance makes it difficult to establish a clear definition of a “normal” or “healthy” gut microbiome (Bäckhed et al., 2012; Wilkins et al., 2019; Oh and Rezaie, 2022). Consequently, identifying dysbiosis becomes subjective since it represents a deviation from an undefined healthy state (Tiffany and Bäumler, 2019).

A stool microbial test, TestUrGut^®^, has been developed to address this challenge. This test consists of qPCR detection of a comprehensive set of 15 microbial markers that represent key functions of the gut microbiota, such as immune protection, mucosal homeostasis, proteolysis, and proinflammatory activity. These markers were selected based on their association with dysbiosis-related disorders and their potential as diagnostic indicators. Additionally, from the analysis of these markers, 2 indices were derived. One is the Bacillota/Bacteroidota index, which is related to diet characteristics (De Filippo et al., 2010) and body mass index (Magne et al., 2020). The other is an indicator of dysbiosis, utilizing the relative abundance of two key microbial species, *Faecalibacterium prausnitzii* and *Escherichia coli*, known to be associated with dysbiosis (Lopez-Siles et al., 2014).

This study aimed to evaluate the clinical utility and validate the representativeness of selected microbial markers and the dysbiosis index. Additionally, this study aimed to distinguish between pathological and transient dysbiosis, contributing to our understanding of the underlying basis of symptomatology related to bowel patterns. Through the evaluation of the clinical utility of the stool microbial test, we aimed to assess the validity of the designed panel of markers, the robustness of the tolerance ranges and the concordance with the dysbiosis index. The results of this study provide valuable insights into the utility of the test in clinical practice and its potential contribution to the understanding and management of specific intestinal disorders.

## 2 Materials and methods

### 2.1 Study population

The sample size of the study (N) was 154. The patients who were recruited previously underwent fecal microbiota tests after being visited by a gastroenterologist due to the presence of digestive discomfort at the NEUPSI-Clínica Bofill Centre in Girona, Spain. Of these 154 patients, 46 were men (29.87%), and 108 were women (70.13%). Clinical data from the enrolled subjects at the time of the examination and their final diagnosis were recorded. The diagnosis was determined by the doctor following usual clinical guidelines in practice.

The inclusion criteria were i) being at least 18 years old, ii) having conducted a TestUrGut^®^ analysis, and iii) having duly signed informed consent. The exclusion criteria were i) having received antibiotic treatment in the last month before collecting the fecal sample, ii) having the feces sampled more than 48 hours before arriving at laboratory facilities, and iii) being pregnant at the time of inclusion.

### 2.2 DNA extraction and qPCR analysis from stool samples

Total DNA was isolated from the fecal samples using the NucleoSpin™ Soil DNA Kit (Macherey-Nagel GmbH & Co., Düren, Germany) according to the manufacturer’s instructions, and the DNA was eluted in a 100 μL final volume.

The abundances of 15 microbial markers representing the main phyla, groups, and genera present in the gut microbiota were analyzed via real-time quantitative polymerase chain reaction (qPCR): *Akkermansia muciniphila* (AKK), Bacteroidota (BAC), *Candida albicans* (CAN), *Clostridium* cluster I (CLO), *Escherichia coli* (ECO), *Enterococcus* sp. (ENT), *Faecalibacterium prausnitzii* (FAE), Bacillota (formerly Firmicutes, FIR), Gammaproteobacteria (GAM), *Lactobacillus* sp. (LAC), *Methanobrevibacter smithii* (MSM), *Roseburia* sp. (ROS), *Ruminococcus* spp. (RUM), *Clostridium* cluster XIV (XIV), and Eubacteria (EUB).

The quantification of AKK, BAC, CAN, CLO, ENT, GAM, FIR, LAC, MSM, ROS, RUM, XIV, and EUB was conducted by preparing single reactions for each biomarker utilizing the GoTaq^®^ qPCR Bryt Master Mix (Promega, Madison, USA). FAE and ECO were quantified in single reactions for each target using the GoTaq^®^ qPCR Probe Master Mix (Promega, Madison, USA). Each reaction consisted of a final volume of 10 µl containing the master mix and between 12 and 20 ng of genomic DNA template. The 16S and 18S rDNA-targeting primers and probes used in this study, along with their respective concentrations, are listed in **Table 1**. These primers and probes were procured from Macrogen (Seoul, South Korea). Accuracy was ensured by running samples in duplicate on the same plate alongside a non-template control reaction and a standard curve, which were included in each qPCR run. The mean of duplicate quantifications was used for data analysis. qPCRs were performed using an AriaDx thermocycler (Agilent Technologies, Santa Clara, CA, USA) under the quality standards of ISO13485.

**Table 1:** Forward (F) and reverse (R) primers and probes (PR) used in this work.

Marker acronym	Primers/ Probe	Sequence 5’→3’	Concentration (nmol/L)	Reference
AKK	F R	CAGCACGTGAAGGTGGGGAC CCTTGCGGTTGGCTTCAGAT	250	(Collado et al., 2007)
BAC	F R	CCGGAWTYATTGGGTTTAAAGGG GGTAAGTTCCTGCGTA	100	(Matsuki et al., 2004)
CAN	F R	CTGATTTATGGGTTCCTGAT GTTGATCAATTGAAGTAGAATC	200	(Bautista-mun et al., 2003)
CLO	F R	CTCAACTTGGGTGCTGCATTT ATTGTAGTACGTGTGTAGCCC	300	(Rekha et al., 2006)
ECO	F R PR	CATGCCGCGTGTATGAAGAA CGGGTAACGTCAATGAGCAAA FAM-TATTAACTTTACTCCCTTCCTCCCCGCTGAA-BHQ1	300	(Lopez-Siles et al., 2014)
			100	
ENT	F R	TACTGACAAACCATTCATGATG AACTTCGTCACCAACGCGAAC	200	(Ke et al., 1999)
FAE	F R PR	TGTAAACTCCTGTTGTTGAGGAAGATAA GCGCTCCCTTTACACCCA FAM-CAAGGAAGTGACGGCTAACTACGTGCCAG-BHQ1	300	(Lopez-Siles et al., 2014)
					250
			FIR		F R	GGCAGCAGTRGGGAATCTTC ACACYTAGYACTCATCGTTT	100	(Mühling et al., 2008)
GAM	F R	TCGTCAGCTCGTGTYGTGA CGTAAGGGC CATGATG	100	(Mühling et al., 2008)
LAC	F R	AGCAGTAGGGAATCTTCCA CGCCACTGGTGTTCYTCCATATA	200	(Payne et al., 2012)
MSM	F R	ACGCAGCTTAAACCACAGTC AAAGACATTGACCCRCGCAT	150	(Ramió-Pujol et al., 2020)
ROS	F R	TACTGCATTGGAAACTGTCG CGGCACCGAAGAGCAAT	125	(Larsen et al., 2010)
RUM	F R	GGCGGCYTRCTGGGCTTT CCAGGTGGATWACTTATTGTGTTAA	250	(Ramirez-farias et al., 2009)
XIV	F R	CGGTACCTGACTAAGAAGC AGTTTYATTCTTGCGAACG	250	(Ramirez-farias et al., 2009)
EUB	F R	ACTCCTACGGGAGGCAGCAGT GTATTACCGCGGCTGCTGGCAC	200	(Lopez-Siles et al., 2014)

All probes were 5’-labelled with FAM (6-carboxyfluorescein) as the reporter dye except for PHGII, in which hexachlorofluorescein (HEX) was used. BHQ1 was used as a quencher dye at the 3’-end for all probes. The base R can be adenine (A) or guanine (G); W can be A or thymidine (T); and Y can be cytosine (C) or T.

The thermal profiles varied based on the specific biomarker being analyzed (**Table 2**). For the probes, a melting curve step was included at the end of each qPCR to verify the expected amplicon size and monitor dimer formation.

**Table 2:** qPCR conditions for each microbial marker.

Microbial markers	Total cycles	Denaturing		Annealing and extension		Melting curve	
		Time (min:s)	T^a^ (°C)	Time (min:s)	T^a^ (°C)	Time (min:s)	T^a^ (°C)
FAE and ECO	40	02:00 10:00	50 95	00:15 01:00	95 60	NA	NA
AKK, BAC, CAN, CLO, ENT, FIR, GAM, LAC, MSM, ROS, RUM, XIV, and EUB	40	10:00	95	00:15 01:00	95 60	01:00 00:30 00:30	95 55 95

NA, not of application.

Once the qPCR results in Ct (threshold cycle) units for each marker were obtained, the data were transformed into relative and total abundance values for statistical analysis.

The total abundance values (A, gene copies per gram of stool) for each marker were calculated using the following equation based on the standard curve included in each qPCR run:


$$A=\frac{\left(\frac{V_{e}}{V_{c}}\right)\cdot 10^{\left(Ct-b\right)/m}}{P\cdot C_{ɡ}}$$

where:

*Ct* is the threshold cycle, *b* is the y-axis intercept on the standard curve, *m* is the slope of the standard curve, *Ve* is the volume of elution of the DNA extract (μl), *Vc* is the volume of the DNA extract loaded in the PCR (μl), *P* is the weight of the stool analytical portion (g), and *Cg* is the number of copies of the 16S or 18S rRNA gene each indicator contains in its genome (**Table 3**).

**Table 3:** Number of copies of 16S and 18S (Cg) for each marker.

Markers acronym	Number of copies of 16S and 18S (Cg)	References
CAN	1	(Pendrak and Roberts, 2011)
MSM	2	(Klappenbach et al., 2001)
AKK	3	(Goux et al., 2018)
ENT	4	(Klappenbach et al., 2001)
BAC, LAC, FIR, ROS, RUM, EUB	5	(Klappenbach et al., 2001; Vásquez et al., 2005; Větrovský and Baldrian, 2013)
GAM	5.5	(Větrovský and Baldrian, 2013)
FAE	6	(Klappenbach et al., 2001)
ECO	7	(Větrovský and Baldrian, 2013; Zemb et al., 2020)
CLO, XIV	8	(Větrovský and Baldrian, 2013)

The number of copies of the phyla Bacteroidoita and Bacillota, as well as the total microbial load (Eubacteria), or markers that include different species, such as Gammaproteobacteria or *Clostridium*, was calculated using an average of the number of copies of the species that form these groups.

Furthermore, each microbial group’s relative abundance was calculated by normalizing the data to the total microbial load (Eubacteria) and subsequently applying a logarithmic transformation to improve data distribution and model fitting. Normalization to total Eubacteria was performed to account for methodological variability related to differences in microbial DNA yield due to variable water content in fecal samples. Despite using a fixed sample weight (40 mg), such variability can affect the total microbial load, and normalization ensures more consistent and comparable results across samples.

### 2.3 Definition of tolerance values

To determine the tolerance range values indicative of a healthy population, as well as those at the borderline and beyond the accepted norms, we conducted an initial analysis on a cohort of healthy individuals. This analysis was subsequently validated in a separate group comprising 24 healthy subjects and 6 patients with digestive diseases, including ulcerative colitis, Crohn’s disease, and irritable bowel syndrome.

Within the healthy cohort, 14 samples were from female subjects, and the age range spanned from 21 to 69 years, with a mean age of 41 years.

The tolerance values were established by calculating the relative abundance of each microbial marker using logarithmic transformations. Specifically, the abundance of each marker was normalized to the abundance of Eubacteria. The mean and standard deviation (SD) of these ratios were then calculated, with the SD either added or subtracted from the mean, depending on the nature of the microbial marker, as a borderline of the accepted norms.

SD was added to markers considered beneficial or protective — AKK, BAC, FAE, FIR, LAC, ROS, RUM, and EUB. Conversely, the SD was subtracted from markers where abundances above established values could be harmful—CAN, ECO, ENT, GAM, and MSM. For the markers CLO and XIV, recognizing that having too little or too much can be detrimental, the SD was both added and subtracted from the mean, resulting in two tolerance limit values above and below the average.

The resulting tolerance values demonstrating limits beyond the accepted norms were obtained by adding or subtracting a unit based on whether the microbial marker was beneficial to the borderline limit of the accepted norms.

Additionally, the relationship between the abundance of Bacillota (FIR) and Bacteroidota (BAC) was calculated by subtracting the logarithm of the FIR from the BAC. No tolerance values were assigned to this index, as its results are indicative of the type of diet rather than dysbiosis.

The dysbiosis index was calculated by logarithmically subtracting the abundance of *Faecalibacterium prausnitzii* from the abundance of *Escherichia coli*, following the approach proposed by Lopez-Siles et al. (Lopez-Siles et al., 2014). This index was originally designed to reflect a microbial imbalance commonly observed in intestinal inflammatory conditions, based on the inverse behavior of these two taxa, representing respectively anti-inflammatory and potentially pro-inflammatory profiles. While other microbial groups, such as Enterococcus, may also be relevant in dysbiotic states, this index was selected for its proven clinical relevance and reproducibility in previous studies.

The average dysbiosis index among healthy controls defined the reference range. Thresholds were then established based on standard deviations from this mean, allowing the classification of individuals into healthy, mild dysbiosis, or severe dysbiosis categories. The present study applies this index to fecal samples, adapting the original calculation—initially based on human cell normalization—to normalization by sample weight, following the approach described in Cusachs (Cusachs, 2021).

### 2.4 Statistical analysis

#### 2.4.1 Data preprocessing

The dataset included several variables, some of which had missing values. To address this, multiple imputation by chained equations (MICE) was applied to estimate missing values based on the available data using the “mice” package in R (Groothuis-Oudshoorn and van, 2011) In this study, five imputations were generated (m = 5), the maximum number of iterations was set to 50 (maxit = 50), and the imputation method was random forest (meth = “rf”). Missing values were limited to four microbial markers (Bacteroidota, Bacillota, Gammaproteobacteria, and *Methanobrevibacter smithii*) in 22 out of 154 samples. These missing values were a consequence of an early change in primer design affecting only a subset of samples. To ensure comparability across the dataset, these values were imputed using the MICE procedure. Consequently, the FIR/BAC balance index—which incorporates these markers—was also imputed in these same 22 samples. The imputed values generated by the MICE algorithm were subsequently used for further analysis.

#### 2.4.2 Statistical approach

For the analysis of the data, a combination of univariate analysis of variance (ANOVA), multivariate analysis of variance (MANOVA), and regression models was used. MANOVA was first used to detect global differences across the full panel of microbial markers in relation to clinical and demographic variables. After MANOVA analysis, follow-up ANOVAs were conducted to identify specific microbial markers contributing to those differences. Markers found to be significant in the univariate analysis were then included in regression models to quantify their association with outcomes of interest. Statistical power calculations were performed using the R package “pwr” to assess whether the sample size was adequate to detect meaningful differences (Champely, 2020). For the group comparisons reported, power values ranged from 96.5% to 100%, indicating that the study was well-powered. Given the limited number of pre-specified comparisons and the consistently high statistical power, no adjustment for multiple comparisons (e.g., Bonferroni correction) was applied.

Regression models are mathematical tools used to establish the relationship between a response variable (Y) and explanatory variables (X). Our study aimed to examine the relationships between the abundance of microbial markers and the occurrence of disease and symptoms.

To accommodate the diverse nature of our response variables, which could be continuous or categorical, generalized linear models (glm) were applied, using a binomial link for binary outcomes (equivalent to logistic regressions). These models are suitable for data with nonnormally distributed errors, which aligns well with the characteristics of our dataset.

The effect size was expressed as an odds ratio (OR), indicating the change in odds of the outcome for each unit increase in the explanatory variable. A value of OR equal to 1 signifies no association between the variables.

All analyses were performed using version 4.1.3 of the R statistical software (R Core Team, 2013).

## 3 Results

### 3.1 Sex

**Figure 1:**
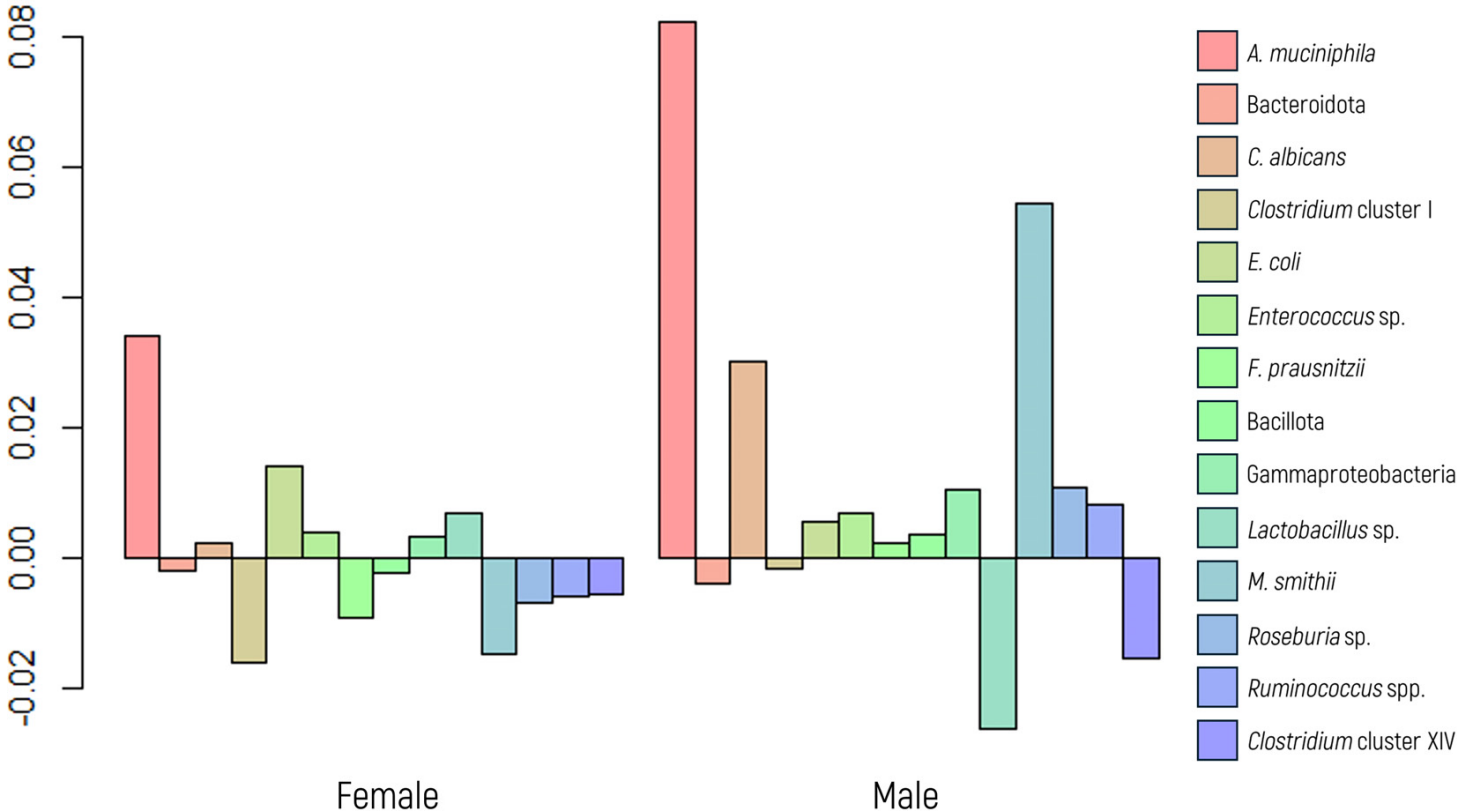
Log-ratio differences in geometric mean abundance (Y axis) of each microbial marker according to sex. Y-axis values represent the natural logarithm of the ratio between groups. A value of 0.05 corresponds to an approximately 5.1% increase in geometric mean abundance.

The MANOVA conducted to investigate the panel’s microbial abundance revealed no statistically significant differences between the sexes (**Figure 1**). However, follow-up ANOVA indicated a significant difference in the abundance of *A. muciniphila* between sexes (p value of 0.0481). This result was further validated using a glm regression model (p value of 0.0104). The OR computed for the *A. muciniphila* was 2.54, indicating that women exhibit a notably greater abundance of this marker than men.

### 3.2 Age

Patients were categorized into three age groups: young individuals (aged 18 to 28, n=12), adults (aged 29 to 59, n=89), and older individuals (aged 60 and above, n=27), following common epidemiological classifications (e.g., WHO, Eurostat) and previous microbiota studies that report age-related differences in gut microbial composition (Odamaki et al., 2016).

**Figure 2:**
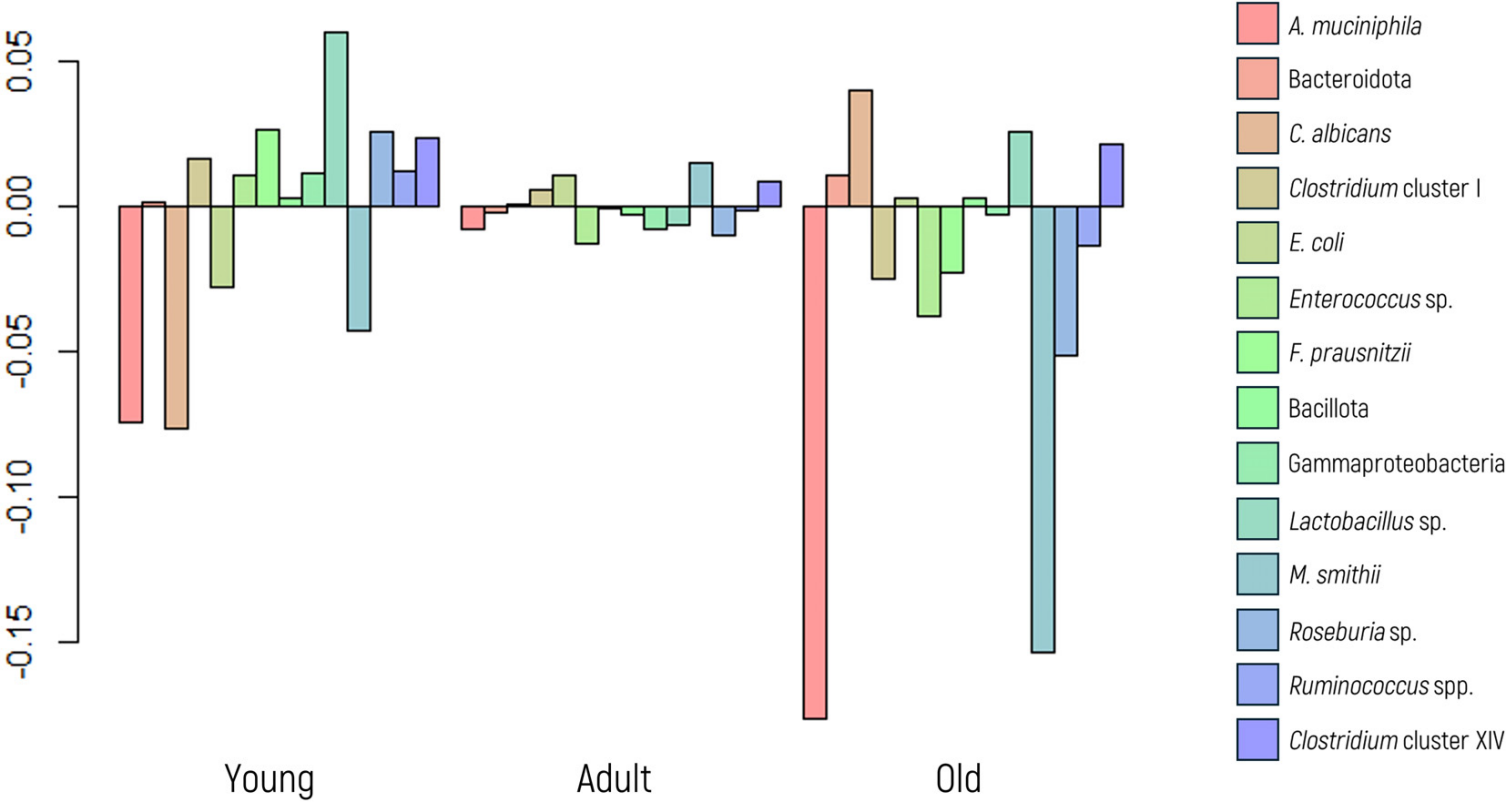
Log-ratio differences in geometric mean abundance (Y axis) of each microbial marker according to age group (young, adult, or older). Y-axis values represent log-ratios between group means.

In this analysis, neither the MANOVA, ANOVA, nor the glm model exhibited statistically significant differences across all microbial markers studied (**Figure 2**). These findings collectively indicate that age does not affect the abundance of any specific microbial marker analyzed.

### 3.3 Body mass index

Body mass index (BMI) data were available for 65 patients, and imputation was not conducted due to a considerable number of missing values. Based on their BMI values, patients were categorized into four groups: underweight (BMI< 18.5, n=4), normal weight (BMI 18.5 to 24.9, n=43), overweight (BMI 25 to 29.9, n=14), and obese (BMI ≥ 30, n=4).

A MANOVA suggested an overall effect of BMI on microbial composition. To explore this further, ANOVA and regression analyses were conducted. *M. smithii* emerged as the microbial marker most influenced by BMI, showing a significant association in the glm model (p value = 0.0147), despite not achieving significance in the ANOVA (p value of 0.1643). Conversely, *A. muciniphila*, a marker extensively linked to BMI in the literature, did not exhibit a significant p value in the glm model; however, it yielded a noteworthy result in the ANOVA (p value of 0.0346).

**Figure 3:**
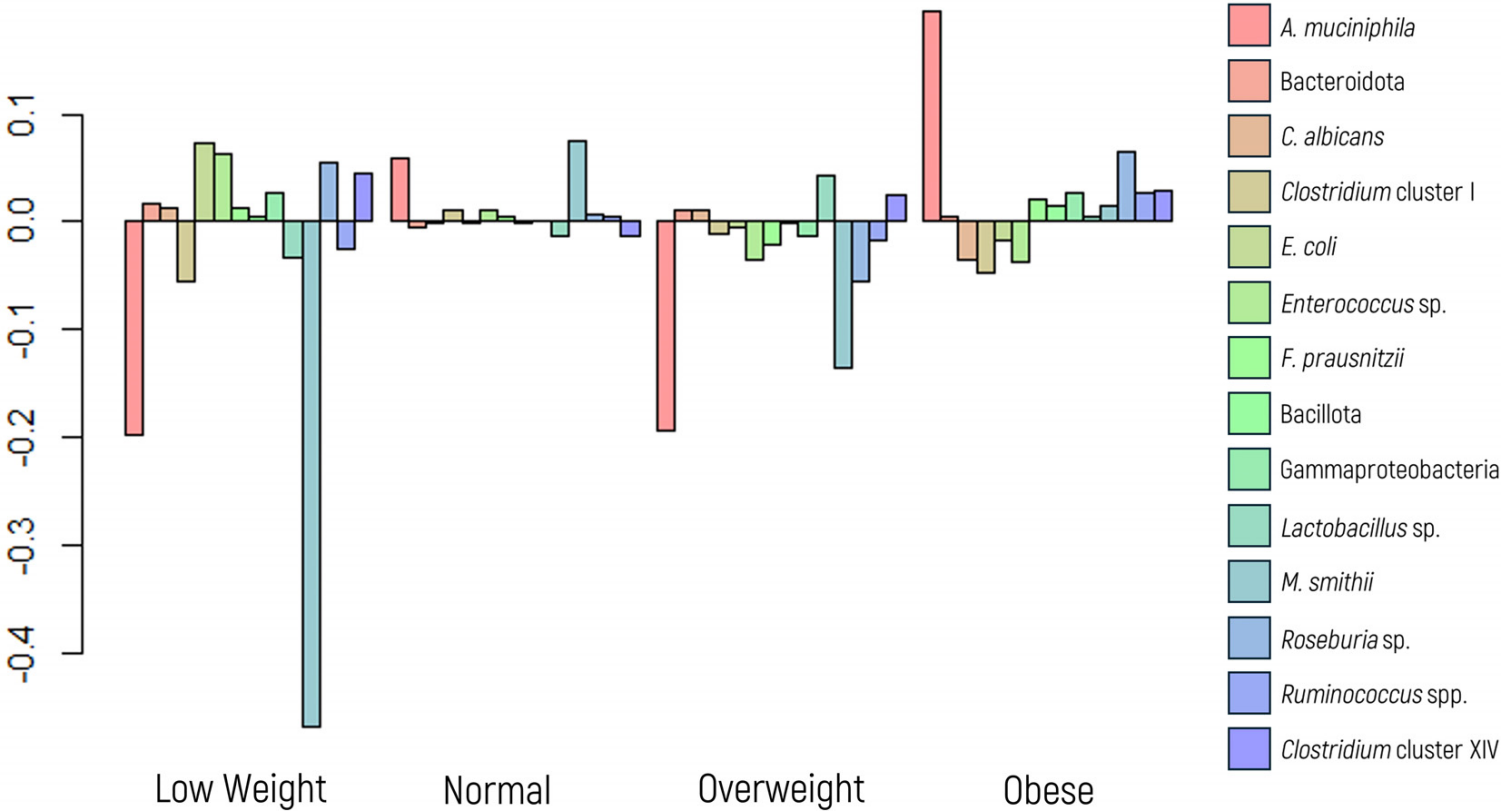
Log-ratio differences in geometric mean abundance (Y axis) of each microbial marker according to body mass index categories: underweight, normal weight, overweight, and obese. Y-axis values reflect log-ratio comparisons.

**Figure 3** illustrates a significant decrease in the abundance of *A. muciniphila* and *M. smithii* in low-weight patients, whereas the first displayed increased abundance in those classified as obese.

### 3.4 Intestinal microbiome profile and digestive symptoms

Among the patients included in this study, 97% were diagnosed with IBS according to the ROME IV criteria, limiting the scope for analyzing the relationship between marker abundance and other diseases, such as mental disorders, neurodegenerative disorders, coeliac disease, and intolerances, among others. As a result, our analysis focused solely on the behavior of IBS.

#### 3.4.1 IBS behavior

Behavior data were available for 62 of the 128 patients who were diagnosed with IBS. Among them, 30 patients exhibited a diarrheal pattern (48.4%), 19 had constipation (30.6%), and 13 (21.0%) presented mixed behavior.

**Figure 4:**
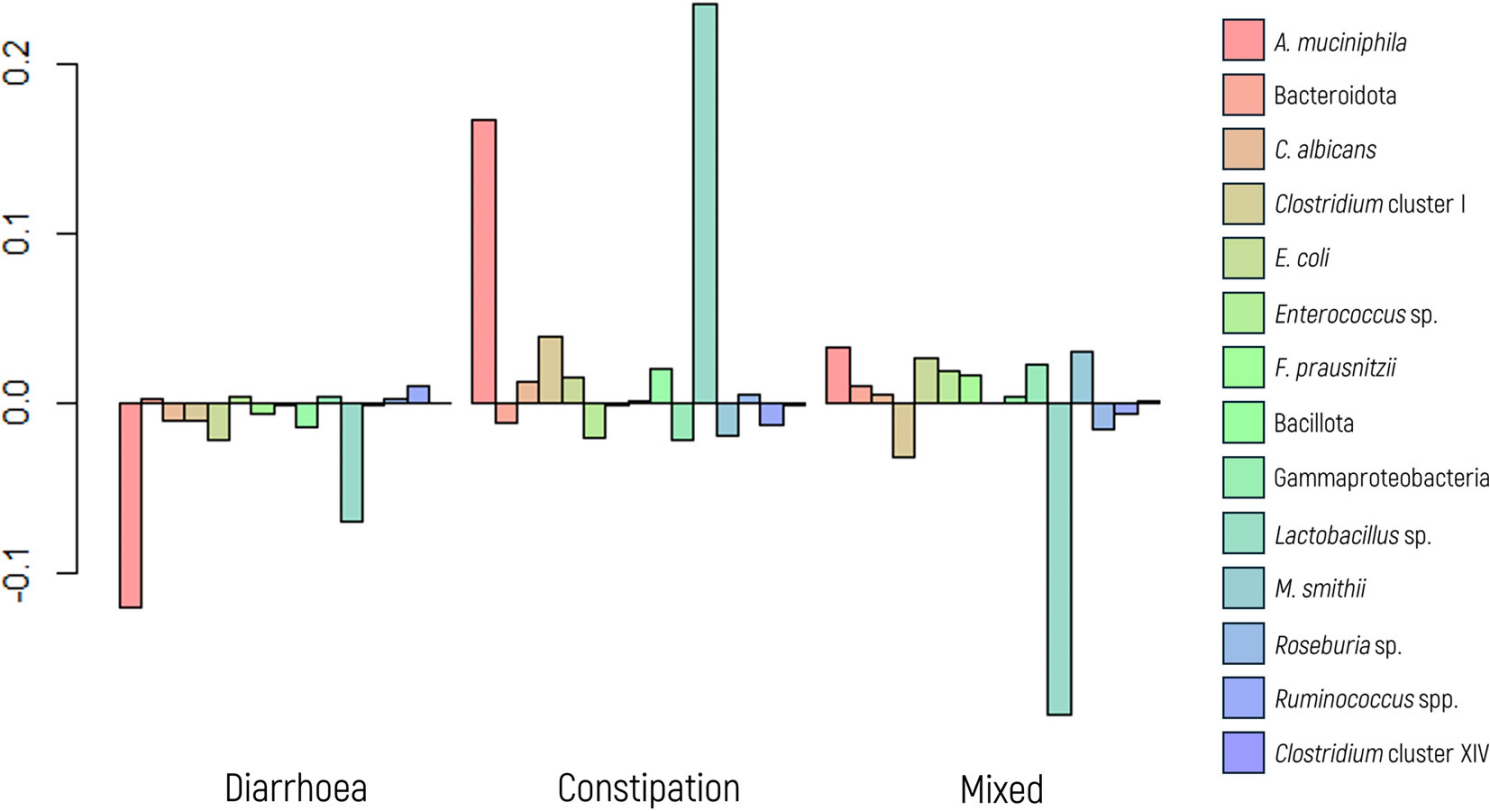
Log-ratio differences in geometric mean abundance (Y axis) of each microbial marker according to irritable bowel syndrome behavior (diarrhea, constipation, and mixed). Y-axis values indicate log-ratios between subgroup means.

A MANOVA indicated significant differences in microbial composition between the subtypes (**Figure 4**). Follow-up ANOVA revealed significant differences for *A. muciniphila* and *M. smithii* (p values of 0.009 and 0.004, respectively). These associations were further explored and confirmed using a glm model.

Specifically, when examining the glm model for diarrheal behavior, *A. muciniphila* displayed a significant association (p value = 0.003) with an OR of 0.220, indicating that the abundance of *A. muciniphila* decreases by a factor of 4.54 (1/0.22 = 4.54) with the presence of diarrhea. Although not statistically significant, a trend was observed (p value of 0.093) for *M. smithii*, with an OR of 0.405, suggesting a 2.5-fold decrease in its abundance in the presence of diarrhea.

Conversely, in the analysis of constipation behavior, both *A. muciniphila* and *M. smithii* exhibited significant associations in the glm model (p values of 0.011 and 0.001, respectively), with corresponding ORs of 3.932 and 5.659, respectively. These findings indicate that the abundance of these markers increases by approximately 4.00- and 5.50-fold, respectively, in the presence of constipation.

For patients displaying a mixed behavior of diarrhea and constipation, the glm model revealed a significant association only for *M. smithii* (p value of 0.049), with an OR of 0.306, indicating a decrease in its abundance by a factor of 3.30.

### 3.5 FIR/BAC index

**Figure 5:**
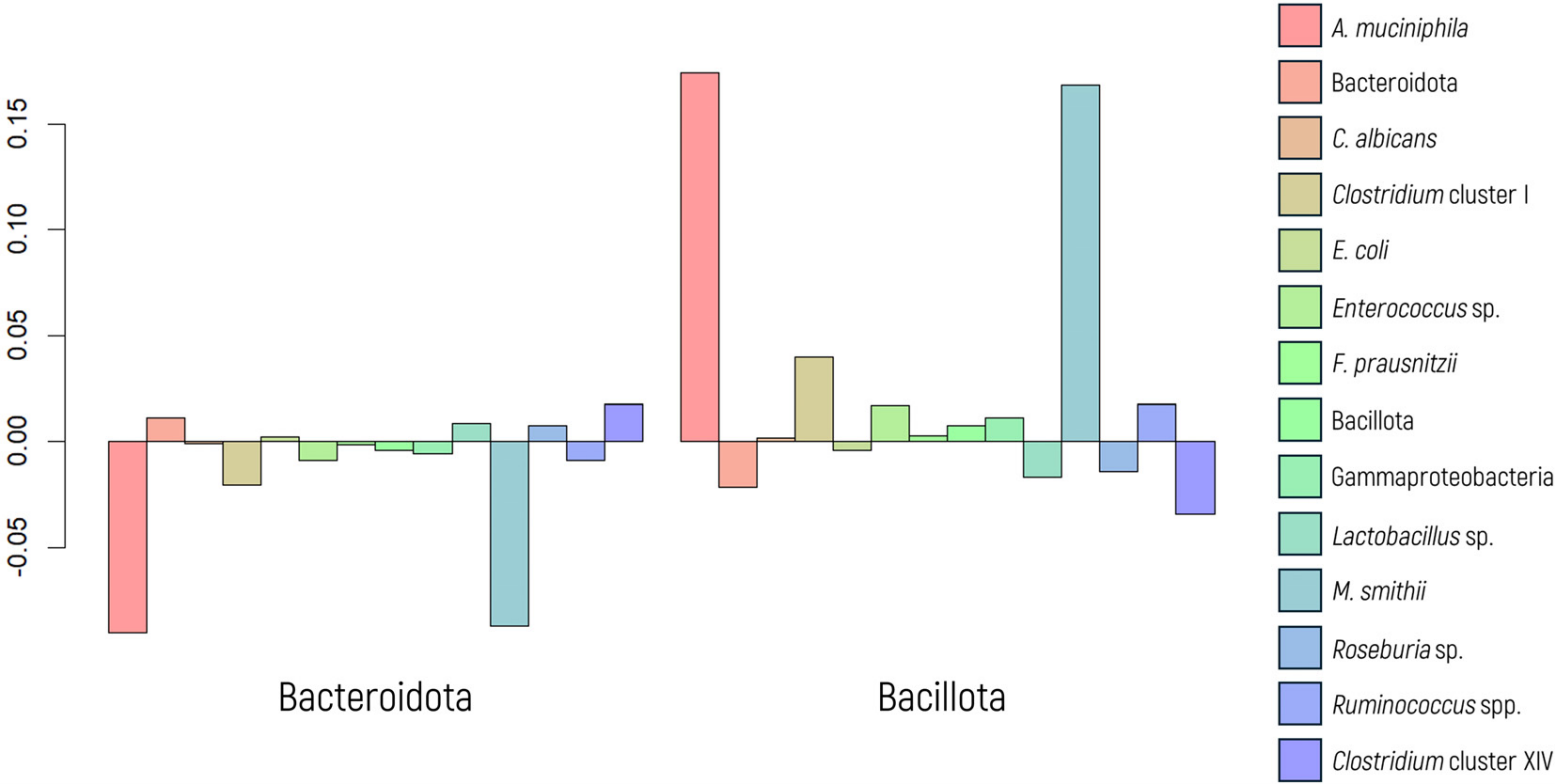
Log-ratio differences in geometric mean abundance (Y axis) of each microbial marker according to Bacillota or Bacteroidota predominance. Y-axis values represent log-ratio comparisons between microbial profiles.

The FIR/BAC index is designated predominantly Bacillota (formerly Firmicutes, FIR) when the index value is positive (greater than zero) and predominantly Bacteroidota (formerly Bacteroidetes, BAC) when the value is negative (less than zero). Significant results were observed in the MANOVA when comparing the abundance of microbial markers based on the predominance of Bacillota or Bacteroidota (p value< 0.001, as depicted in **Figure 5**).

In the MANOVA graph, notable differences in the abundance of *A. muciniphila* and *M. smithii* were evident, as confirmed by ANOVA (p values< 0.001 in both cases). A regression model was employed to assess and quantify this effect, yielding significant values for both markers. The OR indicated a substantial 2.72-fold increase in the abundance of *M. smithii* and a 5.08-fold increase in the abundance of *A. muciniphila* when there was a greater proportion of Bacillota.

**Figure 6:**
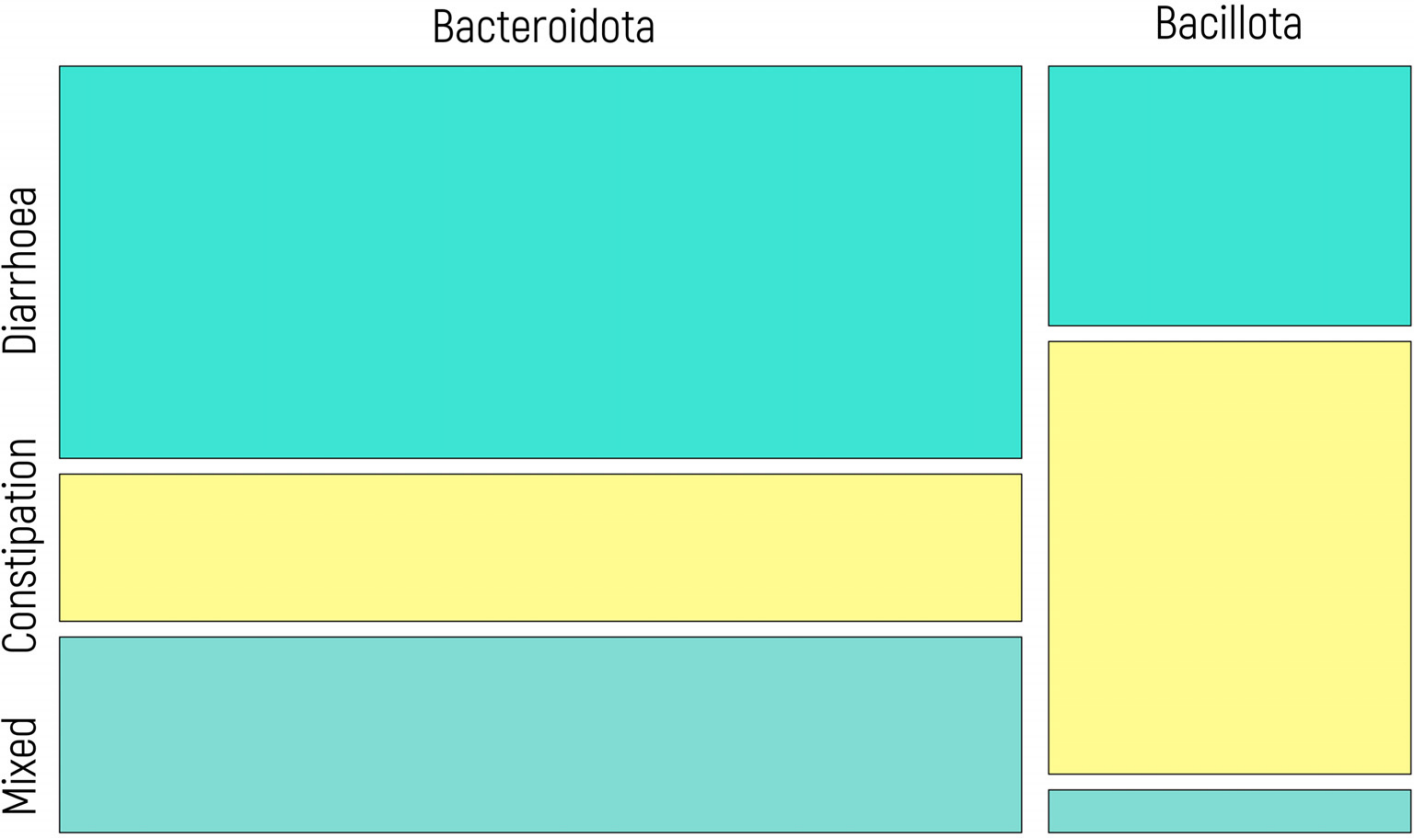
Mosaic plot illustrating the relationship between IBS subtypes (diarrhea, constipation, mixed) and microbial predominance (Bacillota vs. Bacteroidota). The width of each column reflects the proportion of individuals within each IBS subtype, and the height of the colored segments represents the relative frequency of each microbial group within that subtype. Yellow segments correspond to patients with constipation, while blue tones represent patients with diarrhea and mixed behavior. A significant association was observed (chi-square test, p = 0.008).

These findings are closely parallel to those observed in the context of IBS behavior. Consequently, a potential correlation was investigated using a chi-square test, which yielded a statistically significant result (p value of 0.008). **Figure 6** displays a mosaic plot illustrating the relationship between IBS subtypes and microbial predominance. Patients with diarrhea exhibit a greater proportion of Bacteroidota, whereas those with constipation showed a greater prevalence of Bacillota. Patients with mixed IBS behavior exhibit patterns akin to those with diarrhea, with a greater abundance of Bacteroidota.

### 3.6 Dysbiosis index

The dysbiosis index was categorized into three groups (as explained in section 2.3 Definition of tolerance values): “healthy” when the value of the index was greater than 0.66 (n=109) represented values within the healthy range; “mild” values between 0.66 and -0.34 (n=17) indicated a borderline limit slightly decreased index; and “severe” (values< -0.34) (n=5) denoted values well below the healthy range. Significant differences were observed in the MANOVA when comparing the abundance of microbial markers to the dysbiosis index (p value<0.001).

**Figure 7:**
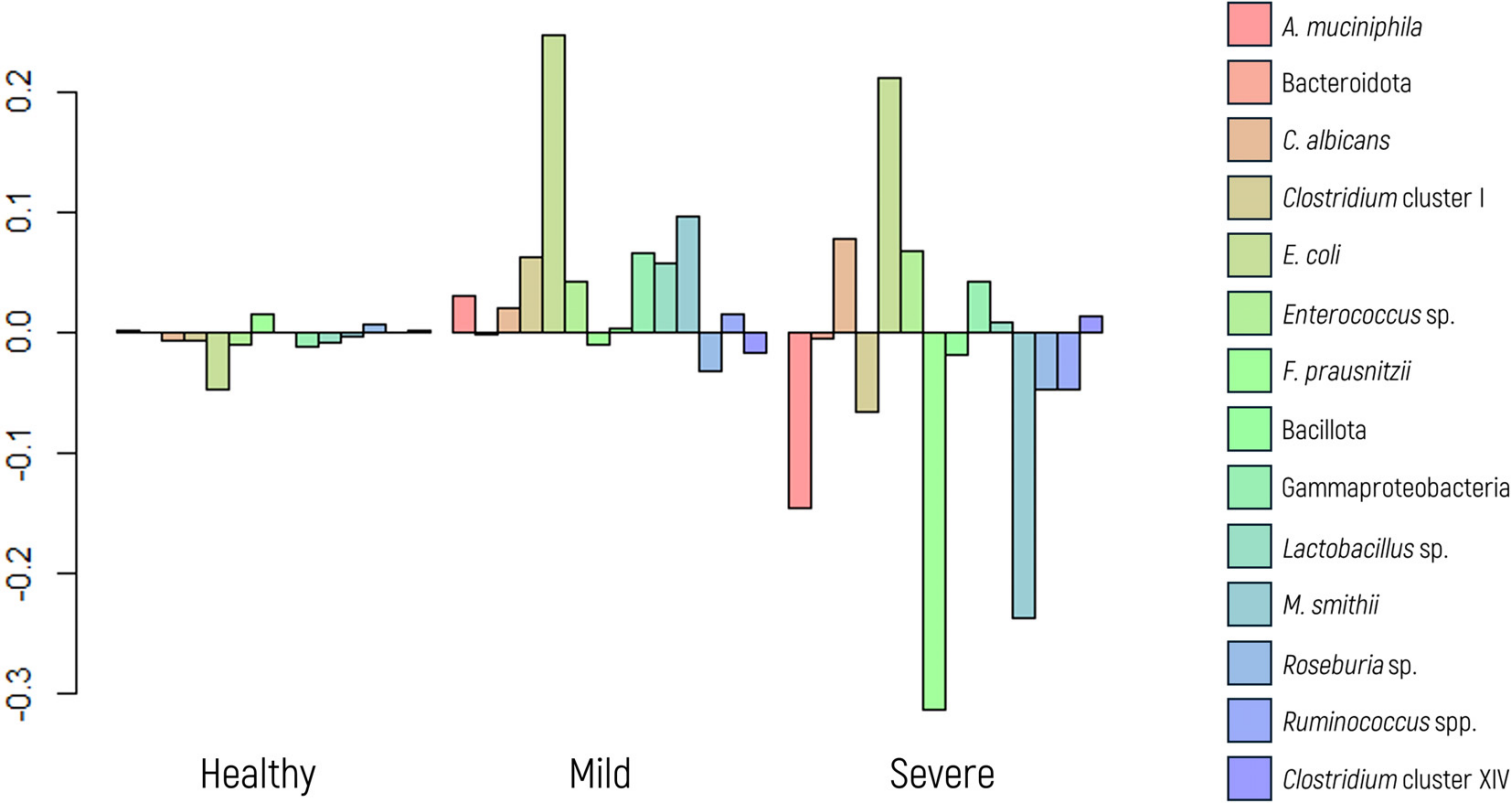
Log-ratio differences in geometric mean abundance (Y axis) of each microbial marker according to dysbiosis category (healthy, mild dysbiosis, or severe dysbiosis). Y-axis values correspond to log-ratios between group means.

Our findings show that a healthy dysbiosis index corresponds to minimal variation in the microbial marker panel (**Figure 7**). Although **Figure 7** presents transformed data for statistical analysis (via ilr transformation), the dysbiosis categories were defined based on non-transformed index values, as detailed in Section 2.3, based on internal validation within the study population. In cases of mild dysbiosis, the variation increases, particularly with a notable increase in some microbial markers. Conversely, severe dysbiosis is characterized by evident imbalances, displaying increased and decreased abundances of microbial markers, notably including decreases in beneficial microbial markers and increases in potential pathogenic species.

Further analysis using ANOVA revealed significant differences in the abundances of specific markers, namely, *Clostridium* cluster I (p value 0.007), and Gammaproteobacteria (p value<0.001). As expected, *E. coli* and *F. prausnitzii* showed significant differences (p value<0.001 in both cases) across dysbiosis categories, given that these taxa were used in defining the dysbiosis index.

## 4 Discussion

In this study, we aimed to evaluate the clinical utility of the novel stool microbial test TestUrGut^®^, which incorporates a set of 15 microbial markers representing critical functions of the gut microbiota. Additionally, two indices were derived: a dysbiosis index derived from the abundance of *Faecalibacterium prausnitzii* and *Escherichia coli*, which was introduced as a quantitative measure to assess microbial imbalances in the gut, and the Firmicutes/Bacteroidetes index, which is related to the type of diet.

Our cohort comprised 154 patients who sought medical consultation for digestive discomfort and who underwent the fecal microbiota test TestUrGut^®^. We categorized the participants based on sex, age, and BMI to explore potential associations with microbial marker abundance and gut dysbiosis.

Significant associations were observed between sex and the *A. muciniphila* marker, with women displaying notably greater abundance than men. *A. muciniphila* is a gram-negative bacterium within the Verrucomicrobia phylum and is characterized as a strict anaerobe capable of producing mucin-degrading enzymes. This bacterium utilizes mucins as a nitrogen and carbon source within the mucus layer of the epithelium. During mucin fermentation, *A. muciniphila* decomposes these substances into acetic and propionic acids and releases sulfate (Xu et al., 2020). The enrichment degree of *A. muciniphila* has been considered an indicator of body metabolic status, encompassing parameters such as glucose homeostasis, serum lipids, and adipocyte distribution in humans (Zhang et al., 2019). Given that human sex exhibits differences in fat distribution, often linked to variations in sex hormone levels (Haro et al., 2016; Yoon and Kim, 2021), it is plausible that the sex-related discrepancies observed in this study concerning *A. muciniphila* abundance may be influenced by how men and women differentially store excess energy and variations in fat body percentages. In addition to fat distribution and sex hormones, other mechanisms such as sex-specific immune responses and host genetic factors have also been proposed to influence *A. muciniphila* abundance and colonization dynamics (Org et al., 2016). These factors may contribute to the observed differences between sexes and warrant further investigation.

In contrast, age did not appear to influence the abundance of any specific microbial marker in our study. Although some previous research has similarly reported minimal age-related effects on gut microbiota composition (Rodríguez et al., 2015; García-Mantrana et al., 2016), other studies have described significant age-associated shifts across the lifespan (Odamaki et al., 2016; Zongxin Ling and Shaochang, 2022). This discrepancy may be due to differences in study design and population characteristics. Our cohort consisted entirely of symptomatic individuals seeking medical consultation, a clinical context that may override more subtle age-related microbial variations typically observed in healthy populations. Furthermore, while the youngest group was relatively small (n = 12), power analysis confirmed that statistical power was adequate (96.5%), supporting the validity of the observed uniformity in microbial marker abundance across age categories.

Regarding BMI, *M. smithii* showed the most significant association with weight status, contradicting previous studies that reported an increase in *M. smithii* in patients with anorexia nervosa (Breton et al., 2019; Camara et al., 2021). One possible explanation for this discrepancy could be the difference in study populations and clinical context. Unlike those previous studies, which focused specifically on diagnosed anorexia, our cohort included individuals with varying digestive symptoms and undetermined nutritional or metabolic status. Additionally, potential confounding factors such as diet composition, metabolic disorders (e.g., insulin resistance), or medication use were not accounted for in this study and may have influenced *M. smithii* abundance (Samuel et al., 2007; Malat et al., 2024). These elements should be considered in future research to better understand the complex interactions between host metabolism, microbiota, and BMI.

Our analysis of patients diagnosed with IBS revealed significant associations between microbial markers and specific bowel behaviors. Specifically, *A. muciniphila* decreased in abundance in individuals with diarrheal behavior, whereas both *A. muciniphila* and *M. smithii* increased in abundance in the presence of constipation. These findings align with prior research demonstrating that methane gas production by *M. smithii* is correlated with slowing intestinal transit, consequently leading to constipation (Kim et al., 2012; Ghoshal et al., 2016). For *A. muciniphila*, Gobert et al. were the first to observe an increased abundance of this marker in patients with IBS and constipation (Gobert et al., 2016). However, the precise relationship between *A. muciniphila* and chronic constipation remains uncertain (Yarullina et al., 2020). Some studies have proposed that *A. muciniphila* induces a decrease in fecal water content through the degradation of intestinal mucin, resulting in impaired intestinal mucosal barrier function (Cao et al., 2017). Conversely, others have reported that an increase in *A. muciniphila* may be associated with stool firmness, making it more prevalent in individuals with slow transit (Vandeputte et al., 2016).

The analysis of the FIR/BAC index, distinguishing patients with a greater proportion of Bacillota or Bacteroidota, revealed associations with *M. smithii* and *A. muciniphila* that closely mirrored the behavioral patterns observed in the analysis of IBS patients. A greater proportion of Bacteroidota was significantly correlated with a diarrheal or mixed pattern, while a greater proportion of Bacillota was associated with constipation. These findings align with previous research, such as Zhuang’s study in 2018 (Zhuang et al., 2018), which reported an increased abundance of Bacteroidota in patients with diarrhea compared to controls, and other studies linked a greater abundance of Bacillota to patients with constipation (Zhu et al., 2014; Youmans et al., 2015).

The dysbiosis index demonstrated its efficacy as a reliable representative of the general dysbiotic state. Furthermore, this study successfully distinguished between varying degrees of dysbiosis severity. Our findings revealed that severe dysbiosis exhibits more pronounced variations in abundance than mild dysbiosis within the specified tolerance values. The congruence between these variations allows for meaningful clinical inferences to be drawn. Specifically, mild dysbiosis is characterized by an increased abundance of potentially pathogenic markers, while severe dysbiosis is associated with decreases in the abundance of beneficial markers together with an increased abundance of potentially pathogenic markers.

Nevertheless, it remains unclear whether the observed alterations in microbial markers are a cause, or a consequence of the symptoms reported. Dysbiosis may result from underlying pathological conditions but may also be modulated by external factors such as dietary patterns, medication use (e.g., antibiotics, proton pump inhibitors), or host physiology (Hooks and O’Malley, 2017; Zmora et al., 2019). Future longitudinal or interventional studies will be essential to better understand the directionality of these associations and to disentangle causal mechanisms from correlation.

This study has several limitations that should be acknowledged. First and foremost, further exploration and validation of these results are imperative to enhance the robustness and generalizability of the findings, primarily by including a larger and more demographically balanced sample size. Although 154 individuals were included, the sample was predominantly female (70.13%), which may limit the extrapolation of results to the general population. Additionally, the vast majority of participants were white, which did not allow for analyses comparing different ethnic groups or for assessing whether microbiota-based differences may vary across ethnicities. As such, the generalizability of our findings to more diverse populations remains limited. Furthermore, various lifestyle and clinical factors known to influence gut microbiota were not controlled for in this study. These include tobacco use, exercise habits, and particularly dietary patterns, medication intake (such as antibiotics, proton pump inhibitors, or laxatives), and presence of metabolic disorders (e.g., insulin resistance). These variables may act as potential confounders in the observed associations and should be addressed in future studies to strengthen causal inference. Digestive symptoms are often interconnected with mental health conditions, such as stress, anxiety, or depression, through the intricate gut–brain axis. Therefore, it would be advantageous to investigate and analyze the potential effects of these psychological factors on gut dysbiosis and symptomatology. By addressing these limitations and incorporating comprehensive analyses of relevant factors, future studies can provide a more thorough understanding of the complex relationships between the gut microbiota, external influences, and human health. This understanding, in turn, may contribute to developing more effective and tailored therapeutic approaches for individuals affected by dysbiosis-related disorders.

In conclusion, our study yields valuable insights into the interplay between gut microbial markers, bowel behaviors, and the dysbiosis and FIR/BAC indices. In this study, our analyses elucidate the utility and representativeness of the selected microbial markers within the TestUrGut^®^ stool microbial test. These markers emerge as robust indicators of the overall state of the microbiota, demonstrating varying abundances intricately linked to the clinical symptomatology observed in patients.

Furthermore, our validation process extends beyond statistical analyses to encompass the translation of results into clinically relevant conclusions, particularly regarding symptomatology and patterns observed in IBS patients. The results of the dysbiosis index reinforce the robustness of our findings with the identification of alterations in the index, along with its correlation to the maximum deviations from the reference values in the panel. This indicates that both the tolerance levels and the index exhibit consistency and clinical utility, thereby providing precise and reliable support for the interpretation and decision-making process. The TestUrGut^®^ stool microbial test is a promising diagnostic tool for discerning gut dysbiosis-related conditions, as the dysbiosis index has been shown to be a good indicator of the status of the overall microbiota. The potential of TestUrGut^®^ extends beyond digestive diseases, encompassing a broad range of conditions previously associated with dysbiosis. Implementing the TestUrGut^®^ test offers a potential avenue for quantifying the intestinal microbiota, advancing towards more targeted and effective interventions. However, it is crucial to further validate and refine the test through additional research, encompassing diverse cohorts and larger sample sizes, to establish its clinical validity and broader applicability.

## Data Availability

The raw data supporting the conclusions of this article will be made available by the authors, without undue reservation.
